# Intrahousehold Transmission of Pandemic (H1N1) 2009 Virus, Victoria, Australia

**DOI:** 10.3201/eid1709.101948

**Published:** 2011-09

**Authors:** Caroline van Gemert, Margaret Hellard, Emma S. McBryde, James Fielding, Tim Spelman, Nasra Higgins, Rosemary Lester, Hassan Vally, Isabel Bergeri

**Affiliations:** Author affiliations: Burnet Institute, Melbourne, Victoria, Australia (C. van Gemert, M. Hellard, E.S. McBryde, T. Spelman, I. Bergeri);; Australian National University, Canberra, Australian Capital Territory, Australia (C. van Gemert, J. Fielding, H. Vally);; Monash University, Melbourne (M. Hellard);; Victorian Department of Health, Melbourne (E.S. McBryde, J. Fielding, N. Higgins, R. Lester);; Royal Melbourne Hospital, Melbourne (E.S. McBryde);; University of Melbourne, Melbourne (E.S. McBryde);; Victorian Infectious Diseases Reference Laboratory, Melbourne (J. Fielding)

**Keywords:** cross-sectional study, pandemic (H1N1) 2009, family characteristics, secondary transmission, quarantine, antiviral prophylaxis, influenza, viruses, Australia, research

## Abstract

To examine intrahousehold secondary transmission of pandemic (H1N1) 2009 virus in households in Victoria, Australia, we conducted a retrospective cross-sectional study in late 2009. We randomly selected case-patients reported during May–June 2009 and their household contacts. Information collected included household characteristics, use of prevention and control measures, and signs and symptoms. Secondary cases were defined as influenza-like illness in household contacts within the specified period. Secondary transmission was identified for 18 of 122 susceptible household contacts. To identify independent predictors of secondary transmission, we developed a model. Risk factors were concurrent quarantine with the household index case-patient, and a protective factor was antiviral prophylaxis. These findings show that timely provision of antiviral prophylaxis to household contacts, particularly when household members are concurrently quarantined during implementation of pandemic management strategies, delays or contains community transmission of pandemic (H1N1) 2009 virus.

## Abstract 2

TOC Summary: Antiviral prophylaxis for quarantined contacts reduces secondary transmission.

Households play a major role in secondary transmission of pandemic influenza. Modeling estimates that household transmission has accounted for 25%–40% of all pandemic (H1N1) 2009 cases (*1**,**2*). Although understanding the effect of individual-level and household-level factors on secondary transmission of pandemic (H1N1) 2009 is paramount to informing population-level prevention strategies, few studies have evaluated household-level risk factors (*3**–**8*).

The Australian Health Management Plan for Pandemic Influenza (AHMPPI), revised in 2008, provides a framework for preparedness and response to pandemic influenza (*9*). The emergence and magnitude of pandemic (H1N1) 2009 in Melbourne, Australia (*10**–**15*), coupled with intensive follow-up and case identification data collected during the delay and contain phases of the AHMPPI (*16*), presented a unique opportunity to characterize intrahousehold transmission during a period of community transmission. Introduction of a suite of prevention and control measures in accordance with AHMPPI also provided an opportunity to measure the effects of these interventions on pandemic (H1N1) 2009 virus transmission.

We therefore conducted a retrospective cross-sectional study of index case-patients and their household contacts in Melbourne (population >3.5 million), Australia (*17*). We examined transmission of pandemic (H1N1) 2009 in households, identified possible risk factors for intrahousehold secondary transmission, and assessed the effects of prevention and control measures introduced to limit transmission.

## Methods

### Participants

The sample population consisted of all persons with confirmed cases of pandemic (H1N1) 2009 reported to the Victorian Department of Health (VDOH) during the delay and contain phases of AHMPPI (May 18–June 3, 2009) from 2 neighboring municipal regions in Melbourne with high numbers of pandemic (H1N1) 2009 notifications. To ensure that only the first reported case in a household could be randomly selected, we flagged households with >1 confirmed case. The index case-patient and household contacts were then recruited by mail and telephone (up to 5 calls were attempted). Of those who could be contacted, we assessed the household’s eligibility according to the Australian Bureau of Statistics definition of a family (households of >2 persons residing together, including at least 1 person <18 years of age, related by blood, marriage, de facto, adoption, or fostering) (*18*).

### Data Collection

During November 18–December 21, 2009, interviewers administered questionnaires to index case-patients and their household contacts. Data collected included demographics, case details, and prevention and control measures used. Participants indicated dates of symptom onset and prevention and control measures used in a retrospective diary of the period of interest (May 11–June 14, 2009). Interpreters were used as requested or needed. A parent or guardian was also interviewed when a participant was <18 years of age. If a household member was not available, a parent, guardian, or partner provided information. Written informed consent was obtained for all participants; parents or legal guardians provided written informed consent for participants <18 years of age.

### Definitions

Index case-patients were defined as patients with the first laboratory-confirmed case of pandemic (H1N1) 2009 in a household reported to the VDOH. Household contacts were defined as persons residing in the same household at the time of the index case-patient’s symptom onset.

Cultural and linguistic diversity was defined as speaking English only or speaking languages other than English in the home. The latter category included those households in which English was a second language.

A secondary case-patient was defined as a household contact who met the case definition for having an influenza-like illness (ILI), defined as self-described fever plus chills and/or respiratory tract signs or symptoms such as cough, sore throat, or shortness of breath with onset 1–9 days after onset for the index case-patient. This interval was based on a serial interval (the number of days between symptom onset in the index case-patient and household contacts) of up to 9 days to identify secondary cases, given that shedding of seasonal influenza virus rarely lasts >8 days (*7*,*19*) and a median incubation period for seasonal influenza of ≈1.4 days (*7*,*20*). Secondary cases were not required to be laboratory confirmed. Household contacts who met our definition for having ILI but who reported symptom onset on the same day as or before that of the index case-patient were not considered to be at risk for secondary transmission and were not included in analysis for exposures associated with secondary transmission.

Use of antiviral drugs (treatment or prophylaxis) was self-reported. VDOH provided antiviral treatment to those who met the case definition (confirmed or suspected case) and whose symptom onset was within 48 hours and provided antiviral prophylaxis to household contacts. Quarantine was self-reported and defined as separation and restriction of movement of case-patients and contacts in their homes (*21*). During the contain phase, patients with confirmed cases were advised to quarantine themselves for 7 days after symptom onset, and contacts were advised to quarantine themselves at home for 7 days after the most recent exposure to an infectious case-patient. A case-patient was considered infectious for 7 days after symptom onset or until acute respiratory symptoms resolved, whichever was longer (*21*).

### Analysis

Chi-square tests were used to determine differences in clinical signs and use of prevention and control measures between index case-patients and household contacts. The Fisher exact test statistic, used to determine nonrandom associations between 2 categorical variables, was used when the expected value was <6. Secondary attack rates (SARs) were calculated by dividing the number of secondary cases by the total number of susceptible household contacts. We stratified SARs for several potential predictors, including individual-level factors, prevention and control measures, and household-level factors. Potential predictors included gender, age group (0–4, 5–19, 20–49, >50 years), relationship to index case-patient (parent/child, sibling, partner, other family member, or other), use of antiviral drugs (treatment or prophylaxis), number of days quarantined with index case-patient, household size (2–3, 4–5, >6 persons), number of children living in the household (1, 2, >3 children), and cultural and linguistic diversity (English only spoken at home and English and/or other languages spoken at home).

Unadjusted logistic regression was used to identify significant candidate predictors (p<0.05) for inclusion in the final adjusted model. The final model used reverse stepwise selection procedures in which all significant predictors of secondary transmission were included in the initial model and removed sequentially until only significant predictors (p<0.05) remained. We accounted for household clustering in the unadjusted and adjusted logistic regression models; that is, we adjusted for dependency of all potential predictors based on membership in the same household by using a generalized estimated equation with robust error estimates, assuming conditional independence within each family (i.e., within the family, each member had independent probability of becoming a case-patient). Goodness of fit for both models was assessed by using the Hosmer–Lemeshow test to 0.05 significance. Statistical analyses were conducted by using Stata version 10 (StataCorp LP, College Station, TX, USA). To indicate precision of the measurement, we have reported 3 significant (i.e., nonzero) figures.

### Ethical Considerations

Participants were reimbursed with $A30. Ethical approval was obtained from the Alfred Hospital Ethics Committee and Australian National University Ethics Committee.

## Results

### Participation and Response Rates

Data extracted on October 20, 2009, contained records for 857 confirmed cases of pandemic (H1N1) 2009, representing 772 households, reported on or before June 3, 2009, including a total of 181 cases for persons residing in the selected municipalities. We then randomly selected 72 case-patients to participate in this study, of which 12 refused, 21 could not be contacted, and 3 did not meet eligibility requirements; the remaining 36 index case-patients and their 131 household contacts participated. Participating and nonparticipating index case-patients were similar in age and student status; however, more nonparticipating (n = 4) than participating (n = 2) index case-patients required an interpreter. Among the 36 households that participated in the study, 32 (88.9%) persons were interviewed face to face and 4 (11.1%) were interviewed by telephone. Interpreters were used for interviews in 2 households.

### Participant Characteristics

The analysis included 36 index case-patients and 131 household contacts (Table 1). The age range of index case-patients was 6–47 years; that of household contacts was 1–74 years. The number of persons living in each household was 2–14, median 4.5 persons. The number of children living in each household was 1–7; most (75.0%) households had 1–2 children. In half of the households (n = 18), a language other than English was spoken at home.

**Table 1:** Characteristics of pandemic (H1N1) 2009 case-patients and household contacts, Victoria, Australia, May 18–June 3, 2009*

Characteristic	No. (%) index case-patients, n = 36	No. (%) household contacts, n = 131	p value
Individual level			
Sex			
M	25 (69.4)	69 (52.7)	0.07
F	11 (30.6)	62 (47.3)	
Age, y			
0–4	0	13 (9.92)	<0.001
5–19	31 (86.1)	40 (30.5)	
20–49	5 (13.9)	68 (51.9)	
>50	0	10 (7.63)	
Household level		NA	NA
No. persons			
2–3	5 (13.9)		
4–5	22 (61.1)		
>6	9 (25.0)		
No. children		NA	NA
1	12 (33.3)		
2	15 (41.7)		
>3	9 (25.0)		
Cultural and linguistic diversity		NA	NA
English only spoken at home	18 (50.0)		
English and/or other language(s) spoken at home	18 (50.0)		

*NA, not applicable.

### Prevention and Control Measures

Antiviral treatment was taken by 30.6% of index case-patients and 4.58% of all household contacts (Table 2). Just under half (45.8%) of all household contacts reported taking antiviral prophylaxis; and among those who did, 1 person reported subsequent symptoms consistent with ILI. The proportion of index case-patients and household contacts who reported being quarantined differed significantly (88.9% and 69.5%, respectively, p = 0.013).

**Table 2:** Prevention and control measures used by pandemic (H1N1) 2009 case-patients and household contacts, Victoria, Australia, May 18–June 3, 2009*

Reported measure	No. (%) index case-patients, n = 36	No. (%) household contacts, n = 131	p value†
Antiviral			
Treatment	11 (30.6)	6 (4.58)	<0.001
Prophylaxis	0	60 (45.8)	<0.001
Quarantine duration, d			
>1	32 (88.9)	91 (69.5)	0.013
>1 with index case-patient	NA	80 (61.1)	

*NA, not applicable. †Fisher exact test statistic used when expected value <6.

The median number of days to initiate quarantine was 3 days for index case-patients and 4 days for household contacts. Greater than half (61.1%) of household contacts reported concurrent quarantine with the index case-patient for at least 1 day; the range of concurrent quarantine was 1–15 days, median 4 days.

**Figure 1:**
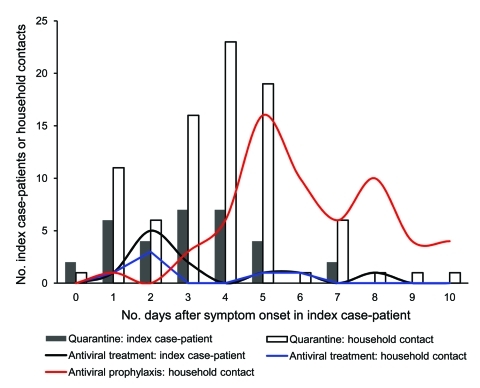
Timeliness of quarantine initiation and administration of antiviral (treatment and prophylaxis) by pandemic (H1N1) 2009 index case-patients and household contacts after onset of symptoms in the index case-patients, Melbourne, Victoria, Australia, May 18–June 3, 2009.

The median number of days before antiviral treatment was initiated for index case-patients and household contacts was 2 days (Figure 1). The median number of days before antiviral prophylaxis was initiated among household contacts was 6 days.

### Clinical Features

**Figure 2:**
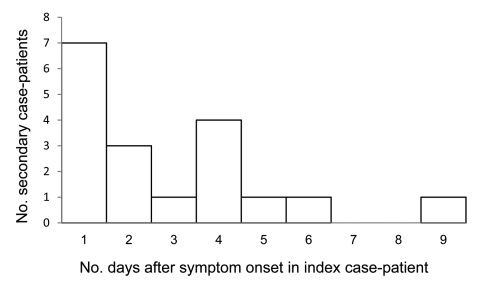
Serial interval for symptom onset in pandemic (H1N1) 2009 index case-patient to symptom onset in secondary case-patients, Melbourne, Victoria, Australia, May 18–June 3, 2009.

Among 131 household contacts, 122 (93.1%) were considered to be at risk for secondary transmission. Among these, 18 reported symptoms consistent with ILI within 1–9 days of symptom onset for the index case-patient and were thus considered secondary case-patients (Figure 2). Household contacts who reported symptom onset before the index case-patient (n = 5), on the same day as the index case-patient (n = 4), or >9 days after onset of symptoms in the index case-patient (n = 3) were not considered to be secondary case-patients and were not included in analyses. The serial interval for secondary cases included in the analysis was 1–9 days, median 2 days.

With the exception of vomiting, clinical features reported by index and secondary case-patients did not differ significantly (range p = 0.275–0.667, Table 3). The most frequent duration of symptoms for index and secondary case-patients was 4–6 days; 31.3% and 37.0% of index and secondary case-patients, respectively, reported symptom duration within this range. Approximately three fourths (77.8%) of secondary case-patients sought medical care (p = 0.01). Prevention or control measures used by index case-patients and secondary case-patients did not differ significantly (quarantine p = 0.429, antiviral prophylaxis p = 0.429, antiviral treatment p = 0.095)

**Table 3:** Clinical features for pandemic (H1N1) 2009 case-patients and household contacts, Victoria, Australia, May 18–June 3, 2009

Feature	No. (%) index case-patients, n = 36	No. (%) secondary case-patients, n = 18	p value*
Sign or symptom			
Fever	35 (97.2)	18 (100)	0.67
Chills	17 (47.2)	8 (44.4)	0.54
Headache	25 (69.4)	13 (72.2)	0.55
Muscle pain	20 (55.6)	8 (44.4)	0.32
Joint pain	15 (41.7)	7 (38.9)	0.54
Fatigue	30 (83.3)	16 (88.9)	0.46
Diarrhea	8 (22.2)	2 (11.1)	0.28
Vomiting	16 (44.4)	2 (11.1)	0.01
Upper respiratory tract symptoms	32 (88.9)	17 (94.4)	0.45
Sign or symptom duration, d			
1–3	9 (25.0)	2 (11.2)	0.49
4–6	13 (36.1)	9 (50.0)	
7–9	9 (25.1)	3 (16.7)	
>10	5 (13.8)	4 (22.2)	
Any medical care received	36 (100)	14 (77.8)	0.01
Reported prevention and control measures taken			
Quarantine	32 (88.9)	15 (83.3)	0.43
Antiviral prophylaxis	0	1 (5.56)	0.43
Antiviral treatment	11 (33.3)	2 (11.1)	0.10

*Fisher exact test statistic used when expected value was <6.

### Secondary Transmission

The overall SAR in this study was 14.8% (95% confidence interval [CI] 8.90%–22.3%, Table 4). The SAR varied when stratified for different individual-level and household-level factors. In unadjusted analysis, predictors of intrahousehold secondary transmission were being female, concurrent quarantine with the index case-patient, and use of antiviral prophylaxis (Table 5). We did not find a significant association between secondary case-patients and age group, relationship to the index case, household size, number of children living in the household, or cultural and linguistic diversity. In the adjusted analysis, p value for gender decreased from 0.037 to 0.83 and was thus removed from the final model. In the final model, the odds of a household contact who was concurrently quarantined with the index case-patient becoming a secondary case-patient increased for each additional day (adjusted odds ratio 1.25, 95% CI 1.06–1.47), and the odds of secondary transmission among household contacts who reported use of antiviral prophylaxis decreased (adjusted odds ratio 0.042, 95% CI 0.004–0.434). We did not identify a significant interaction term to include in the multivariate model.

**Table 4:** Secondary attack rates for susceptible household contacts of index case-patients with pandemic (H1N1) 2009, Victoria, Australia, May 18–June 3, 2009*

Variable	Total no. household contacts	No. with influenza-like illness	Secondary attack rate, % (95% CI)
Individual-level associations			
Sex			
M	58	5	8.62 (1.08–14.4)
F	64	13	20.3 (11.3–32.2)
Age, y			
0–4	11	1	9.09 (0.230–41.3)
5–19	35	6	17.1 (6.50–33.6)
20–49	66	10	15.2 (7.51–26.1)
>50	10	1	10.0 (0.25–44.5)
Relationship to index case-patient			
Parent/child/partner	65	10	15.4 (7.63–26.5)
Sibling	44	8	18.2 (8.19–32.7)
Other family member	13	0	0 (0–24.7)
Prevention and control measures reported			
Antiviral prophylaxis	57	1	1.8 (0.04–9.39)
Quarantined >1 d with index case-patient	73	15	20.5 (12.0–31.6)
Household-level associations			
No. persons			
2–3	7	2	28.6 (3.67–71.0)
4–5	75	10	13.3 (6.58–23.2)
>6	40	6	15.0 (5.71–29.8)
No. children			
1	31	6	19.4 (7.45–37.5)
2	47	7	14.9 (6.20–28.3)
>3	44	5	11.4 (3.79–24.6)
Cultural and linguistic diversity			
Only English spoken at home	53	5	9.4 (3.13–20.7)
English and/or other language(s) spoken at home	69	13	18.8 (10.4–30.1)

*CI, confidence interval.

**Table 5:** Unadjusted associations with secondary transmission for pandemic (H1N1) 2009, Victoria, Australia, May 18–June 3, 2009*

Variable	OR (95% CI)	p value
Individual level		
Sex		
M	1.00	
F	2.70 (1.060–6.860)	0.037
Age, y		
0–4	1.00	
5–19	2.06 (0.179–23.90)	0.560
20–49	1.79 (0.228–14.00)	0.581
>50	1.11 (0.529–23.30)	0.946
Relationship to index case-patient		
Parent/child/partner	1.00	
Sibling	1.22 (0.562–2.660)	0.613
Other family member	†	
Reported prevention and control measures		
Antiviral prophylaxis‡	0.05 (0.006–0.429)	0.006
Quarantined for >1 d with index case-patient§	1.22 (1.03–1.44)	0.019
Household level		
No. persons		
2–3	1.00	
4–5	0.385 (0.035–4.280)	0.437
>6	0.441 (0.024–8.070)	0.581
No. children		
1	1.00	
2	0.729 (0.163–3.260)	0.679
>3	0.534 (0.05–5.74)	0.605
Cultural and linguistic diversity		
Only English spoken at home	1.00	
English and/or other language(s) spoken at home	2.23 (0.448–11.100)	0.328

*Backwards stepwise selection procedures were used to develop the final adjusted model whereby predictors (p>0.05) were removed sequentially until only significant predictors (p<0.05) remained. Gender was not significant in the adjusted model (p = 0.83) and was thus removed. Goodness of fit for both models was assessed by using the Hosmer and Lemeshow test to 0.05 significance. Goodness of fit for the final model was 0.2. OR, odds ratio; CI, confidence interval. †No secondary cases occurred in this group, and this level is not included in the unadjusted model. ‡Adjusted OR 0.042 (95% CI 0.004–0.434); p = 0.008. §Logistic regression using number of days quarantined with index case-patient as continuous exposure. Adjusted OR 1.25 (95% CI 1.06–1.47); p = 0.008.

## Discussion

This study fully characterizes transmission of pandemic (H1N1) 2009 in households in Australia during implementation of pandemic management strategies to delay or contain community transmission. The findings are relevant for prevention and control strategies used at the household level indicated in the AHMPPI and for international pandemic influenza planning. Overall, 14.8% of susceptible household contacts became secondary case-patients, assumed to have been infected by the index case-patient. The SAR for ILI observed in this study is within the range of reported SARs for ILI used as a proxy for pandemic (H1N1) 2009 in similar international studies, which were 3.7%– 45% (*4**–**8**,**22**–**27*).

The odds of seeking medical care were lower for secondary than for index case-patients. Although this finding was expected because of the case ascertainment methods used, other factors involved with health care–seeking behavior should be considered. For example, household contacts may have not sought care because VDOH provided antiviral treatment and prophylaxis to household contacts without requiring evidence of laboratory-confirmed disease. Furthermore, symptomatic household contacts may have reasonably assumed that they were infected with pandemic (H1N1) 2009 given their proximity to a confirmed case-patient and may not have considered confirmation necessary. The differences in health care–seeking behavior have implications for the pandemic influenza response, particularly during the phases of the AHMPPI when emphasis is on active case finding and slowing community transmission. This finding highlights the need for timely household-level, rather than individual-level, provision of treatment and prevention strategies by health care professionals, at the point of care of the index case-patient.

Several individual-level and household-level factors influenced the SAR and the odds of secondary transmission within households. The odds of becoming a secondary case-patient were almost 3× greater for female than male contacts, possibly because more women assume caregiver roles and therefore having a greater likelihood of exposure. This explanation is supported by France et al. (*4*), who reported that providing care to a case-patient was associated with a higher risk for ILI among parents. A study with greater power may be able to demonstrate this association in adjusted analyses. Other studies have also reported findings that older age was protective against secondary transmission of pandemic (H1N1) 2009, possibly as a result of prior immunity in older age groups (*4**,**5*). Although a decreasing trend of secondary transmission was observed for participants 5–19 years to 20–49 years of age, the size of this study was insufficiently powered to demonstrate a significant association between age group and rate of secondary transmission.

Our finding that antiviral prophylaxis reduced the odds of secondary transmission by 95% among at-risk household contacts was greater than that reported by France et al., who reported a 68% reduction in risk (*4*). Although this finding highlights the potential for antiviral prophylaxis to prevent secondary transmission, it should be considered along with the finding that initiation of antiviral treatment and prophylaxis for index case-patients and household contacts was considerably delayed. Current evidence highlights that rapid implementation of prevention measures such as antiviral prophylaxis is critical for control of pandemic influenza as soon as community transmission is identified; our findings identify an area for improvement in the implementation of pandemic influenza management plans. For example, the need for timely use of antiviral prophylaxis was demonstrated by Donnelly et al., who found that only 18% of pandemic influenza transmission events take place >2 days after onset of symptoms in case-patients (*28*). Ghani et al. also demonstrated this need when they reported a 3-fold increase in odds of intrahousehold secondary transmission in households that did not receive antiviral prophylaxis within 3 days of index case-patient symptom onset (*2*). Similarly, Goldstein et al. report that early antiviral treatment (on the day of or day after symptom onset) reduced the odds of household secondary transmission by 42% (*29*).

The issue of timeliness was also identified with regard to initiation of quarantine. We identified a considerable delay between onset of symptoms in the index case-patient and initiation of quarantine for index case-patients and household contacts, thus prolonging community exposure to pandemic (H1N1) 2009. Quarantine of case-patients and close contacts is considered an essential strategy for mitigating community transmission of pandemic influenza (*9*); however, to reduce the rate of community transmission, case-patients need to be quarantined as early as possible during their infectious period.

Although quarantine has been demonstrated to be effective at reducing community attack rates in pandemic influenza modeling studies, it has been hypothesized that the subsequent increase in contact rates between household members during quarantine may increase intrahousehold transmission (*30*). We found evidence supporting this hypothesis, demonstrating that the odds of secondary transmission increased >20% for each additional day of quarantine with the index case-patient. Similar effects of quarantine on intrahousehold secondary attack rates have not been reported for pandemic (H1N1) 2009; however, a study of university students in the People’s Republic of China found an increased attack rate among contacts who shared a room or bathroom with confirmed pandemic (H1N1) 2009 case-patients (*31*), and a study in New York reported increased risk between siblings who interacted closely with the index case-patient (*4*). Thus, to prevent community transmission, effective communication to confirmed case-patients as well as their household contacts to ensure timely implementation of quarantine measures is needed. This finding should be considered along with previously discussed public health implications, including the recommendation for implementation of prevention and control measures at the household level rather than the individual level to ensure that messages reach household contacts. Furthermore, to counter the increased risk associated with quarantine with the index case-patient, quarantine should be implemented concurrently with distribution of antiviral prophylaxis to household contacts.

The influence of cultural and linguistic diversity on secondary transmission served as a proxy for a range of social and environmental determinants of intrahousehold transmission of pandemic (H1N1) 2009 transmission, including recognition and understanding of health promotion messages and access to antiviral treatment and prophylaxis during the containment stages of the AHMPPI. A key finding was a higher SAR among persons who spoke languages other than English at home. This finding suggests that control and prevention measures were not effectively communicated, comprehended, and adhered to by a major community subset in Victoria. Although a higher SAR was observed among persons who spoke languages other than English at home, the study had insufficient power to provide evidence for the relative contribution of cultural and linguistic diversity on secondary transmission. Nonetheless, the potential issues associated with effective communication, comprehension, and adherence to prevention and control measures by cultural and linguistically diverse communities suggest that further work should explore the social and cultural determinants of pandemic (H1N1) 2009.

This study has some limitations. First, it was subject to recall bias, which we attempted to reduce by using tools to improve accurate recall of illness (such as case notification information from VDOH and calendars of major events that occurred during the period of interest). Second, information bias may have been introduced by household members who provided information for household contacts not available at the time of interview. This bias occurred during a few interviews; however, any information bias is likely to underestimate the true association between exposures and pandemic (H1N1) 2009. Third, ILI was used as an indicator for pandemic (H1N1) 2009, and thus some misclassification may have occurred. However, because sentinel surveillance indicated that most respiratory infections during the same period were pandemic (H1N1) 2009, misclassification was probably minimal (*32*). Fourth, recruitment of households on the basis of the confirmed status of 1 household member may introduce selection bias; however, during the study period, rates of testing of persons with mild to severe illness were high, and thus household contacts should be representative of influenza infections in the community. Fifth, the sample size was small; nonetheless, we identified several factors significantly associated with secondary transmission of pandemic (H1N1) 2009. Sixth, some ILI might be community acquired and therefore overestimate the rate of secondary transmission; we attempted to mitigate any overestimation by excluding concurrent primary cases and household contacts who reported symptom onset before that of the index case-patient.

Our study findings can aid the continued development of future pandemic influenza preparedness plans in Australia and internationally. In particular, the provision of treatment and prevention strategies at the household level, rather than at the individual level alone at the point of care of the index case-patient, should be considered. The need for engagement at the household rather than the individual level is further emphasized by the benefit of timely provision of antiviral prophylaxis to household contacts, particularly when household contacts are quarantined concurrently with the index case-patient. The integration of these practical findings in the development of pandemic influenza preparedness plans in Australia and internationally can help reduce the potential for intrahousehold transmission of influenza during future pandemics.
